# Slowly learned but not forgotten: New learning in a case of childhood‐acquired amnesia

**DOI:** 10.1111/jnp.12423

**Published:** 2025-03-06

**Authors:** Kata Pauly‐Takacs, Chris J. A. Moulin

**Affiliations:** ^1^ School of Humanities and Social Sciences Leeds Beckett University Leeds UK; ^2^ Laboratoire de Psychologie et NeuroCognition Institute Universitaire de France, Université Grenoble Alpes Grenoble France

**Keywords:** childhood brain tumours, episodic memory, memory, source memory

## Abstract

This case report presents new semantic learning and long‐term retention data collected over a 5‐year period from an amnesic adolescent boy, CJ. Compared to his younger sister, a novel abbreviation‐learning task captured CJ's slower semantic acquisition across three weekly training sessions. By contrast, his rate of forgetting between sessions was comparable to that of the control's and was slower over long delays of up to 5 years but recalled information without any reliable report of the original learning context.

## INTRODUCTION

Episodic memory deficits in childhood are typically caused by illness or injury that implicates the hippocampus; for example, hypoxic brain injury (i.e. caused by oxygen deficiency), cerebrovascular accidents, viral infections, epilepsy, traumatic brain injury and brain tumours. The most compelling cases of childhood‐acquired amnesia were first described by Vargha‐Khadem et al. (1997). This influential paper presents three young patients who acquired focal bilateral hippocampal injury very early in life (two of them at birth). Interestingly, when these children were tested as adolescents or young adults, it was revealed that they acquired normal levels of intelligence, literacy and general knowledge despite substantially underdeveloped hippocampi, suggesting that the development of their semantic memory was not affected by hippocampal pathology. However, they presented with a profound episodic memory impairment in that they had difficulty remembering specific events both in laboratory and everyday life settings.

The term ‘developmental amnesia’ was originally introduced to describe cases such as those above (Vargha‐Khadem et al., 2001) and implied that only very early acquired (i.e. perinatal or first year of life) and only selective hippocampal pathology would lead to the observed pattern of impaired episodic but preserved semantic memory. However, a later study demonstrated that hippocampal pathology acquired in middle childhood and even early adolescence led to effectively the same memory profile as those who sustained the brain injury very early in life (Vargha‐Khadem et al., 2003). Furthermore, other researchers have since reported functionally similar memory profiles in childhood‐acquired aetiologies that are typically associated with more diffuse neuropathology, for example, in paediatric brain tumours (Vicari et al., 2007; Pauly‐Takacs et al., 2011).

A consistent observation among children who acquired hippocampal injury and consequent episodic memory deficits is that they are able to lay down semantic facts in the real world (e.g. personal facts, such as the name of their new school or names of people), but their rate of new semantic learning is far from normal under laboratory conditions (e.g. Elward et al., 2024; Gardiner et al., 2008; Guillery‐Girard et al., 2004). The amount of new semantic information these children can learn and retain for long‐term retrieval depends on various tasks and individual characteristics. Yet, a common finding among a handful of studies is that even if learning is impaired and slow, requiring more repetition, children with developmental amnesia show little sign of long‐term forgetting, in contrast to their healthy peers who do tend to forget some of the information (see Elward & Vargha‐Khadem, 2018 for a detailed review). Nevertheless, it is unclear just how durable these memories are since long‐term follow‐up tests did not span years after the learning phase in previous studies. Another important observation came from a single case study with Jon, the most studied case of developmental amnesia; while he was able to acquire novel facts, his source judgements for these facts were inaccurate (Gardiner et al., 2008). *Source memory* refers to the various features of memories that give rise to their episodic character, including, for example, the spatial and temporal context of an event (Mitchell & Johnson, 2009) and is therefore conceptually aligned with Tulving's (1985) episodic memory definition, emphasising the contextually embedded nature of experiences.

We had the rare opportunity to work closely with an adolescent boy with childhood‐acquired amnesia, CJ, over a 5‐year period, between 13 and 18 years of the patient's age. Based on our observations, we were motivated to explore new semantic learning and retention over an extended period alongside source memory attributions using materials that we knew CJ would engage with and enjoy. At a school meeting, CJ once asked what GCSE stood for, which inspired the development of our ‘abbreviation learning’ task, described below.

## METHODS

### Case description

CJ's case history and neuropsychological profile has been described in detail elsewhere, but in brief, this previously highly academically able child (the smartest boy in class according to a classmate) was diagnosed with multiple brain tumours at the age of 11 requiring radiotherapy and chemotherapy treatment (Pauly‐Takacs et al., 2011; Pauly‐Takacs & Moulin, 2020). Post‐treatment MRI scan reports noted a marked and generalised cerebral atrophy with associated white matter loss as well as bilateral volume loss to the hippocampus. The illness left CJ with a pronounced episodic memory impairment in the context of some other cognitive difficulties, but general knowledge, semantic processing and language skills were remarkably well preserved. CJ was unable to accurately report on the day‐to‐day events of his life, for example, what lessons he had at school, though he was at times able to respond correctly to questions about happenings of the recent past. Yet, he would comment that he just ‘worked it out’ rather than remembered. CJ achieved good marks in some subjects in school, though his teachers' impression was that this reflected his strong premorbid knowledge base, rather than new learning. While CJ remained enthusiastic about novel concepts in newly introduced subjects, learning and retaining knowledge became a real challenge for him.

### Materials and procedure

We used 12 three‐letter abbreviations as materials, the meaning of which CJ did not know previously. Our primary aim was to track CJ's learning across multiple training sessions 1 week apart (until learning of all abbreviations is achieved), with five trials in each session, and test his retention of new semantic information at different long‐term delay intervals over a 5‐year period. Our secondary aim was to gain insight into CJ's memory for the context of the learning experience by probing the source of knowledge. CJ was 15 years old at the start of the study, and his 11‐year‐old sister (EJ) acted as a control participant. Informed consent and parental assent were obtained. The task was administered by the first author.

CJ and EJ took part in the study individually. The abbreviations were presented visually on printed cards one at a time with their meaning read out loud by the experimenter. For example, ‘NCP stands for National Car Parks’. Immediately after presentation, participants were asked to repeat the meaning of the abbreviation. After the initial presentation of all 12 abbreviations, five further trials followed, each beginning with testing retention by means of cued recall (i.e. by presenting the printed card and the three letters spelt out by the experimenter). If the meaning was recalled verbally in full (i.e. correct response provided for each of the three letters), no further training was provided. If the abbreviation was not recalled or partially recalled, the correct answer was provided verbally again, and participants were asked to repeat it verbally. Abbreviation cards were presented in a different order across the five trials and no breaks were given between trials. Each session lasted between half an hour to an hour depending on the amount of repetition required. Separated by 1 week, each further training session began with testing retention of information learned on the previous week by means of cued recall, as described above. Abbreviations recalled correctly in full were excluded from further training in the session, but it continued for the other items. To test long‐term retention, six follow‐up tests were administered across 5 years once learning of all items was achieved. Follow‐up sessions were test only with the same cued‐recall method and no further training was provided at or in between follow‐ups. Parents confirmed that no further practice of the materials took place at home between training sessions and during the long‐term follow‐up period.

## RESULTS

**FIGURE 1:**
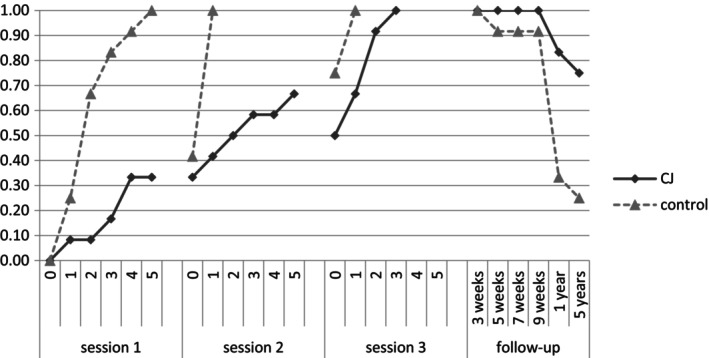
Graph showing the learning curve of CJ and his sister on 12 novel three‐letter abbreviations. Data points in Trial 0 in Sessions 2 and 3 represent retention of information from the previous session a week earlier. Each further trial in training sessions began with a retention test, and further training was provided where necessary for a maximum of five trials. Long‐term follow‐up sessions were test only.

The pattern of learning and long‐term retention of CJ and EJ is shown in Figure 1.

CJ's rate of learning and forgetting during training was markedly different from that of his younger sister's. In general, CJ learned fewer items within a training session, but forgot proportionally less information between sessions 1 and 2 (the siblings' retention between sessions 2 and 3 was identically 75%). CJ needed far more repetition of information overall during training sessions to achieve 100% learning by the end of session 3, while his sister required little further training in sessions 2 and 3 before performance returned to ceiling. CJ's long‐term retention was very good, having recalled all abbreviations in full at follow‐up test sessions covering a 9‐week period; this was better than his younger sister's memory for the abbreviations which decreased to 92%. Striking differences in retention unravelled after longer‐term delays. CJ recalled 10 (83%) and 9 (75%) of the abbreviations in full without hesitation after 1 and 5 years, respectively, while EJ's performance dropped to three items (25%) 5 years later.

In an attempt to capture CJ's memory for the learning context, the siblings were also probed with open‐ended questions as to the source of their knowledge of the abbreviations week by week as part of the training and at most of the long‐term follow‐up sessions (i.e. Where do you know this from? How long have you known this for?). At follow‐up 2 and 4, source memory was probed with a two‐alternative forced‐choice task (i.e. Did you learn this from me? Or did you learn this elsewhere?). Unsurprisingly, for CJ's typically developing sister such questions were easily answered even 5 years after training. Even for abbreviations she had been unable to recall, EJ commented *I know we did these*, indicating that elements of the experience of being taught by the researcher was retained in the absence of retaining the semantic facts. It is CJ's response pattern and style that we found most interesting and thought‐provoking from a theoretical perspective.

In contrast with his sister, it was extremely rare for CJ to attribute knowledge of the abbreviations to the training task carried out with the first author of this paper (see Tables 1 and 2 for a summary).

**TABLE 1:** Proportions of correct source answers by CJ for correct recalls. At follow‐up 2 and 4, source memory was probed using a two‐alternative forced choice test; at the remaining time points, an open‐ended question was used. Follow‐up tests took place 1 = 3 weeks, 2 = 5 weeks, 3 = 7 weeks, 4 = 9 weeks, 5 = 1 year, 6 = 5 years after training.

	Training sessions		Long‐term follow‐up tests					
	2	3	1	2	3	4	5	6
Source CJ	.50	.00	.25	.50	.16	.50	.10	.00

**TABLE 2:** CJ's verbatim source answers for the abbreviations, as reported by him upon the first successful recall.

Abbreviation	Source	When answer was given
NCP	I know it from going to the car park. I know this for 9 years	Follow‐up 1
HST	You told me that a few months ago	Training 2
SMS	I know that from mobiles, for 2 years	Follow‐up 1
WHO	From going to the hospital, I just worked it out. I know it since I was 9	Training 3
GDP	I just know that sort of thing from reading books	Follow‐up 1
IMF	I know it from watching the ‘Weakest link’. I know this for 3 years	Follow‐up 1
EEC	I know it from reading for about 3 years	Follow‐up 1
NPG	I know it from the art gallery, from looking at the pictures a few months ago	Training 2
APR	I know it from going to the bank with mum. I know this for 6 years	Follow‐up 1
VAT	I know it from the bank. I learned this a few weeks ago	Training 2
TOT	I know this from you, last time you came, a few weeks ago	Training 2
FOK	I know it from reading books. I have known this for 6 years	Training 3

Abbreviations: APR, annual percentage rate; EEC, European Economic Community; FOK, feeling of knowing (the latter two abbreviations are derived from metacognition research); GDP, gross domestic product; HST, high speed train; IMF, International Monetary Fund; NCP, National Car Parks; NPG, National Portrait Gallery; SMS, short message system; TOT, tip of tongue; VAT, value added tax; WHO, World Health Organization.

Even when provided two alternatives (at follow‐up 2 and 4), a response rate of 50% correct could be achieved merely by guessing based on binomial probabilities. Instead, consistent with the notion of provoked confabulations, his tendency was to report various alternative, but plausible learning contexts whether it was during the training phase or as part of the long‐term follow‐up tests. His source attributions were qualitatively indistinguishable from abbreviations that he had known previously^1^ and his responses for the same abbreviation were inconsistent across test sessions. Yet, it is not to say that CJ had no awareness or the ability to infer that some of these abbreviations might have been learned from the researcher. On the rare occasion, for some items, he explicitly noted that *You told me that* or that *I have learnt this on these cards with you*. Five years after training, he reported the second author as the source of his knowledge for 50% of the novel abbreviations, although Chris Moulin was never present in these sessions. Over time, training from the researcher itself has become a plausible and reportable source of knowledge in the context of the assessment, but it is unlikely that his correct source attributions represent mental time travel and thus accurate episodic recall.

## DISCUSSION AND CONCLUSION

In sharing these data, we were keen to demonstrate how adequate learning and long‐term retention can be achieved even in a child with profound episodic memory failure. This information is retained well even after a retention interval of 5 years, and CJ outperforms his younger sister. While our data do not speak to the mechanisms involved in supporting CJ's impressive long‐term retention, repetition of information and the application of a training procedure that minimises interference from guessing appears to be key. The neural bases of successful semantic information retention in childhood‐acquired amnesia are not well understood, but it has been argued by others that early acquired hippocampal injury might optimise semantic memory development such that qualitatively different mnemonic processes, those not reliant on episodic memory, are utilised when learning new semantic information (Elward & Vargha‐Khadem, 2018). It may be the case that the cost of such neural compensation is the slowness of learning at the benefit of better long‐term retention. This would explain the opposite pattern of learning and retention in healthy children who do have the capacity to rely on episodic memory in the same task, as demonstrated in our study and previous work. Indeed, the most striking aspect of our data is the lack of any reliable source information, even though this was an easily answered task for the control participant. We might conclude that CJ's learning and retention was achieved without any knowledge of the context of learning the information, even when a logical guess at why the items were being tested was because they had been taught by the experimenter.

It is acknowledged that the use of ecologically valid learning materials for tracking retention over long periods means that our results might be confounded by potential extra‐experimental exposure to those materials. Therefore, it will be important for future studies to include adequately matched healthy participants as controls and consider including children with a similar clinical and educational history who do not take part in the learning phase but whose knowledge of the materials is tested at follow‐ups.

These data are of interest to the neuroscience of memory community because they are a neat example of possible dissociations between remembering information and its context, and the difference between the acquisition and long‐term retention of information. CJ's profile is, on the one hand, impressive since he retains very well what he so difficultly acquired, but also because he is completely unaware of the source of this acquisition. Such findings might be expected according to leading theory regarding the hierarchically organised nature of memory (Mishkin et al., 1997) and add to the body of literature demonstrating new semantic learning with ecologically valid materials but with long retention intervals that have not been previously reported.

## AUTHOR CONTRIBUTIONS

**Kata Pauly‐Takacs:** Investigation; methodology; visualization; writing – original draft; conceptualization; formal analysis. **Chris J. A. Moulin:** Supervision; writing – review and editing.

## CONFLICT OF INTEREST STATEMENT

The authors declare no conflicts of interest.

## Data Availability

The data that support the findings of this study are available upon request from the corresponding author. The data are not publicly available due to privacy or ethical restrictions.

## Footnotes

^1^
Previously known abbreviations were established as part of a pre‐test before training on the 12 novel abbreviations began.
